# Computerized Cognitive Behavior Therapy for Anxiety and Depression in Rural Areas: A Systematic Review

**DOI:** 10.2196/jmir.4145

**Published:** 2015-06-05

**Authors:** Kari Dee Vallury, Martin Jones, Chloe Oosterbroek

**Affiliations:** ^1^ Department of Rural Health (DRH) Division of Health Sciences University of South Australia Whyalla Norrie Australia

**Keywords:** eHealth, mHealth, depression, anxiety, cognitive therapy, rural health, mental health

## Abstract

**Background:**

People living in rural and remote communities have greater difficulty accessing mental health services and evidence-based therapies, such as cognitive behavior therapy (CBT), than their urban counterparts. Computerized CBT (CCBT) can be used to effectively treat depression and anxiety and may be particularly useful in rural settings where there are a lack of suitably trained practitioners.

**Objective:**

To systematically review the global evidence regarding the clinical effectiveness and acceptability of CCBT interventions for anxiety and/or depression for people living in rural and remote locations.

**Methods:**

We searched seven online databases: Medline, Embase Classic and Embase, PsycINFO, CINAHL, Web of Science, Scopus, and the Cochrane Library. We also hand searched reference lists, Internet search engines, and trial protocols. Two stages of selection were undertaken. In the first, the three authors screened citations. Studies were retained if they reported the efficacy, effectiveness or acceptability of CCBT for depression and/or anxiety disorders, were peer reviewed, and written in English. The qualitative data analysis software, NVivo 10, was then used to run automated text searches for the word “rural,” its synonyms, and stemmed words. All studies identified were read in full and were included in the study if they measured or meaningfully discussed the efficacy or acceptability of CCBT among rural participants.

**Results:**

A total of 2594 studies were identified, of which 11 met the selection criteria and were included in the review. The studies that disaggregated efficacy data by location of participant reported that CCBT was equally effective for rural and urban participants. Rural location was found to both positively and negatively predict adherence across studies. CCBT may be more acceptable among rural than urban participants—studies to date showed that rural participants were less likely to want more face-to-face contact with a practitioner and found that computerized delivery addressed confidentiality concerns.

**Conclusions:**

CCBT can be effective for addressing depression and anxiety and is acceptable among rural participants. Further work is required to confirm these results across a wider range of countries, and to determine the most feasible model of CCBT delivery, in partnership with people who live and work in rural and remote communities.

## Introduction

### Background

In any one year, 10% and 14% of Australian adults experience affective problems and anxiety disorders, respectively [1,2]. These rates of mental illness are in line with global trends—the average 12-month depression prevalence rate of 18 high- and low-income countries is 5.4% [3]. For anxiety disorders, 12-month prevalence rates range from 7% to 15.5% in Euro/Anglo cultures [4].

In Australia, over 30% of the population lives outside major cities, with 11% living in outer regional, remote, and very remote areas [5]. The reported prevalence of mental health disorders is similar across rural and urban areas [6,7]. However, there are certain population groups in rural and remote areas that experience higher levels of mental disorders—men in outer regional and remote areas are significantly more likely to experience higher levels of psychological distress than men in major cities [8], and women in nonmetropolitan areas aged 30 to 44 years also face slightly higher rates of mental health disorders than their urban counterparts [6].

Globally, the prevalence of anxiety disorders is significantly higher among rural versus urban populations [4]. Furthermore, suicide rates are markedly higher in rural areas compared with major cities, as has been documented in Australia, the United States, the United Kingdom, and New Zealand [9]. In Australia, suicide rates increase with level of remoteness and are largely driven by increased suicides among young men [6,10,11].

### Treatment, Services, and Access

There is a strong evidence base and subsequently established guidelines for the effective drug and nondrug treatment of depression and anxiety. For example, supportive clinical care, cognitive behavior therapy (CBT), antidepressants, and interpersonal therapy (IPT) are all recommended treatment options for different forms of depression [12]. For anxiety, evidence-based interventions include self-help strategies, group and individual psychoeducational interventions including CBT, and pharmacological treatments for complex conditions [13].

However, despite an understanding of what works in treating depression and anxiety, many people do not receive adequate care. Less than one-quarter of Australians access psychosocial services, even when they are available [14]. Of the people with depression or anxiety who do seek treatment, under half are offered an appropriate treatment option [15].

Accessing health services can be particularly difficult for people living outside metropolitan areas and away from service hubs. Smaller proportions of rural versus urban populations seek or receive professional help for a mental health problem [6,16].

There are numerous factors that prevent people from accessing mental health services. Availability of services and trained mental health professionals are major barriers to access in rural and remote Australia [14,17]. In comparison with the 115 psychologists for every 100,000 persons in major cities, the rate in rural areas declines from 66.5 in inner regional to 29 in very remote areas [18]. Other barriers may include cultural norms around stoicism and not wanting to show vulnerability, denial, poor mental health literacy, stigma around mental illness and mental health service use, and the financial and personal demands required of treatment [6,17,19]. Studies from a number of English-speaking countries have shown that mental health stigma is particularly prevalent in rural areas, is greater among men, and impacts willingness to seek help [16].

The consequences of untreated depression and anxiety are wide ranging and often debilitating. These conditions can lead to reduced quality of life and productivity, increased likelihood of developing substance abuse disorders [20], nonadherence to care and treatment [21,22], increased risk of physical health problems such as cardiovascular disease [23], and increased suicide risk [24]. Stack [24] reported that 87% of suicides involve at least one mental disorder, and that people experiencing major depression are as much as 20 times more likely to commit suicide than people without depression. Accessing appropriate treatment for depression can reduce suicide risk by up to 50%, particularly among young men [12].

### Computerized Cognitive Behavior Therapy

Computerized cognitive behavior therapy (CCBT) is an effective treatment option for people with anxiety and/or depression, both as a standalone treatment and as a component of a stepped-care treatment plan. Numerous reviews and meta-analyses have found that CCBT achieves moderate to large effect sizes for depression and anxiety [25-31], similar to those found for therapist-delivered CBT [26,32]. That said, the comparative effectiveness of CCBT and therapist-delivered CBT is somewhat contested. For example, a Cochrane review by Mayo-Wilson and Montgomery [28] found therapist-delivered CBT to be more effective than computerized delivery.

The delivery of evidence-based psychotherapy via personal computers, mobile phones, and tablets provides an opportunity to increase its uptake in rural and remote communities. It may help minimize the impact of inadequate numbers and unequal distribution of appropriately trained therapists, and subsequent long wait times, as well as the financial demands of treatment, travel times [19,33], and stigma associated with accessing mental health services. Computerized CBT as a mechanism to improve evidence-based service provision in rural and remote areas may increase the uptake of evidence-based interventions. However, few studies have explicitly explored the effectiveness and acceptability of CCBT in rural communities.

This review synthesizes the global evidence regarding the clinical effectiveness and acceptability of CCBT interventions for preventing or treating anxiety and depression in people who live in rural and remote areas. It provides recommendations for future research and practice with relevance to rural communities in English-speaking countries around the world.

## Methods

### Overview

Literature was systematically reviewed in line with the Preferred Reporting Items for Systematic Review and Meta-Analysis (PRISMA) guidelines [34].

### Search Strategy

The search strategy was developed by authors KV and CO with input from an academic research librarian. The final strategy included variations of the following terms: anxiety, depression, eHealth, computerized, online, application, health, cognitive behavior therapy, and computerized cognitive behavior therapy. Other terms such as "e-therapy," "Internet-delivered," and "phone-based" did not identify additional citations and were excluded from the strategy. A full copy of the search strategy is provided in Multimedia Appendix 1.

The search was conducted in a number of health and science databases: Medline (1946-2014), Embase Classic and Embase (1947-2014), PsycINFO (1806-2014), CINAHL (1981-2014), Web of Science (1950-2014), Scopus (1960-2014), and the Cochrane Library (all reviews and trials, May 2014). Additional articles were identified through pearling (ie, hand searching) selected reference lists and trial protocols.

### Selection Criteria

Two screening phases were conducted. In the initial phase, studies were included if they (1) reported the clinical efficacy, effectiveness, acceptability and/or feasibility of CBT delivered via the Internet, through the use of a computer or other mobile electronic device; (2) had a focus on the prevention or treatment of generalized or social anxiety disorders, multiple forms of anxiety, and/or depression; (3) included participants from any population group in any location; (4) were conducted in any year up until the search date of May 22, 2014; and (5) were written in English. Primary and secondary studies with quantitative and qualitative designs, as well as systematic reviews and meta-analyses, were included.

Studies that focused solely on individual phobias, posttraumatic stress disorder, or postnatal depression were not included in this review, although studies that addressed any combination of anxiety disorders were included. Generalized anxiety disorder (GAD) and social anxiety disorder are two of the most prevalent anxiety disorders, with 12-month prevalence rates in Australia of 2.7% and 4.7%, respectively [15]. In the United Kingdom, approximately 4.4% of the population are experiencing generalized anxiety at any one time, in comparison to panic disorder and obsessive compulsive disorder (OCD) at 1.1% [35]. Furthermore, these forms of anxiety are more likely to be treatable with more generalizable forms of CBT. For these reasons, findings regarding the efficacy of CCBT for these disorders are likely to be more broadly applicable and were, therefore, included as the focus of this review.

In the second phase of screening, studies that measured efficacy or acceptability among rural participants or meaningfully discussed the application of CCBT in rural settings were retained for inclusion.

### Study Selection

All three authors were involved in the initial stage of the study selection process. KV conducted an initial review of all citations by title and discarded any that were clearly irrelevant. KV and CO then reviewed the abstracts of all remaining citations (half each), discarding any that did not meet the inclusion criteria. In response to any uncertainty, the other reviewing author was consulted. If both authors were unsure or disagreed, the third author (MJ) was consulted to reach a final decision. Full texts were located for all citations that potentially matched the inclusion criteria. Each text was reviewed by KV and CO independently to decide on the final list of included articles, again with input from MJ when required.

NVivo 10 (QSR International, Cambridge, MA), a software package that supports qualitative data analysis, was used to support the second stage of screening. All studies that met the inclusion criteria at stage one were imported into NVivo. Automated text (word) searches were run to identify studies that included the word “rural,” its stemmed variations, and synonyms. The full texts of studies identified through this process were then assessed by KV to determine the final list of included studies. Where KV was undecided, MJ was consulted.

### Data Extraction and Bias Assessment

A structured, but flexible, data extraction table was developed. Data were extracted for a range of outcomes measuring patient experience as well as clinical effect. These included study design, population and intervention characteristics, clinical efficacy, rates of uptake and adherence, qualitative measures of satisfaction, and perceived benefits and disadvantages.

The Cochrane Collaboration’s tool for assessing risk of bias [36] was used to assess bias among randomized controlled trials (RCTs), assessed at study level*.* Relevant criteria from the Grades of Recommendation, Assessment, Development, and Evaluation (GRADE) handbook were used to assess bias in all other study designs [37]. Several authors were contacted with requests for further information regarding study methods to support the accurate completion of these assessments.

## Results

### Overview

**Figure 1:**
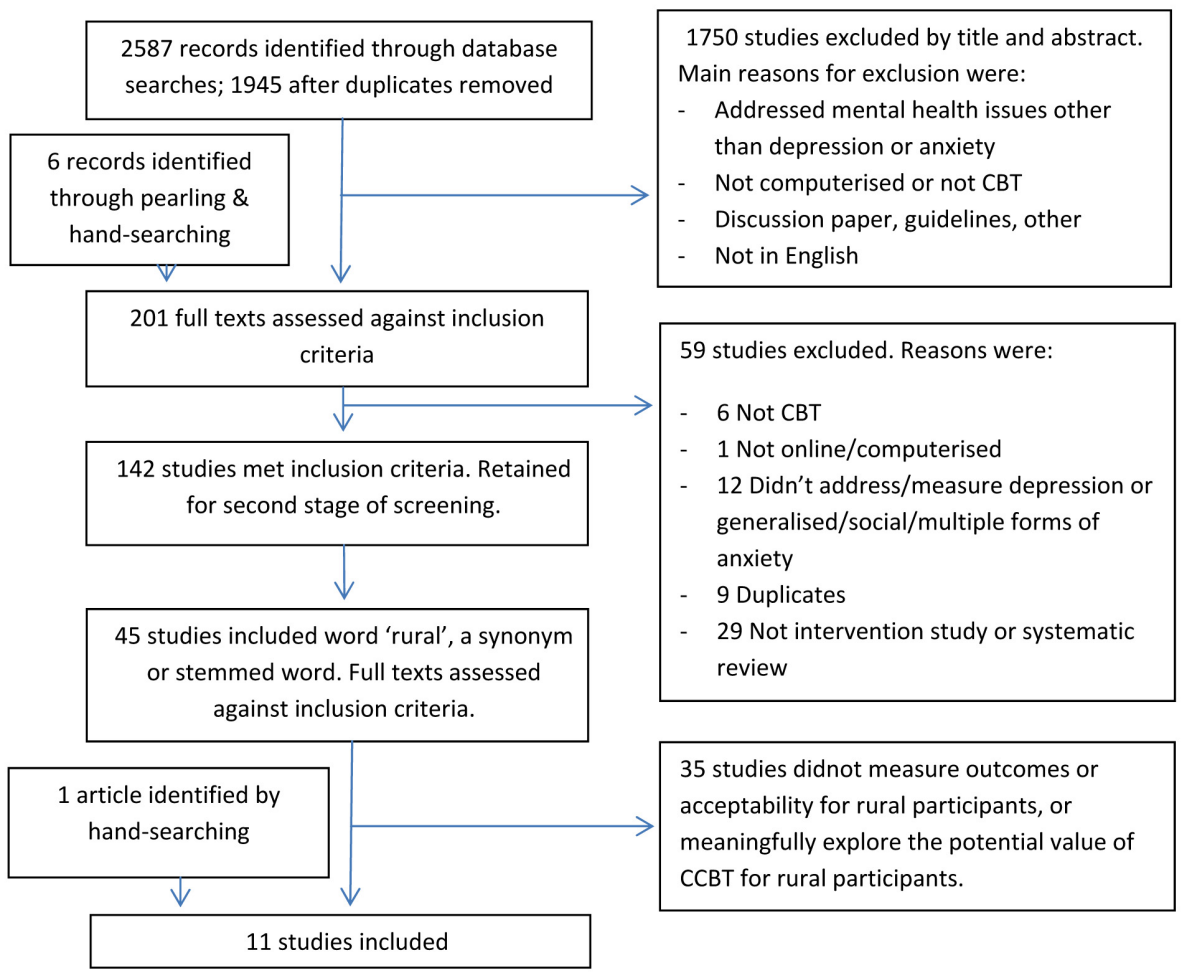
Study selection process.

The initial database search identified 2587 citations. Of these, 195 were selected for full-text review at the first stage of screening, along with six studies identified through pearling. A total of 142 studies met the inclusion criteria and were retained for the second stage of screening. The automated text search revealed that 45 of these studies included the word “rural,” a synonym, or stemmed word at least once. Of these, 10 met the inclusion criteria and were included in the review. One extra study was identified by hand searching at this stage, resulting in 11 studies being finally included in this review [38-48]. Figure 1 outlines this process and provides the reasons for exclusion at each stage.

### Study Characteristics

Among the 11 included studies were four papers reporting the findings of three RCTs, one systematic review, one qualitative study, and five studies which used quasi-experimental designs. Nine of the studies were conducted in Australia and two—regarding one trial—in Scotland. Characteristics and key findings of the 11 studies are reported in Table 1.

Eight papers regarding six different trials measured rural location as a predictor of outcomes, adherence, or acceptability [38-45]. The qualitative study explored the acceptability of a CCBT package among rural youth [46]. The final two studies discussed the potential application of their results to rural populations [47,48].

Across the nine studies reporting at least some rural participants were a total of 11,260 participants. Between 16% and 100% of study participants lived in rural areas. Four of the 11 studies explored the value of CCBT among children and/or young people, while five studies tested CCBT for adults. The systematic review included five studies with adult participants and four with children and/or young people.

**Table 1:** Characteristics of included studies.

Citation; program	Study design	Location	Participants: n, % rural; gender, %; age group, age in years, mean or range	Main findings
Calear et al 2013 [38]; MoodGYM, anxiety & depression	RCT^a^	Australia	1477, ~16; female, 56; adolescents, 12-17	Living in a rural location predicted greater adherence.
Neil et al 2009 [43]; MoodGYM, anxiety & depression	Quasi-experimental	Australia	8207, 19; female, 71; adolescents, 13-19	Living in a rural area predicted greater adherence.
Sethi 2013 [47]; MoodGYM, anxiety & depression	RCT	Australia	89, 0; female, 58; youth, 18-25	CCBT^b^may be a viable option for youth, but unsuitable for people with low literacy.
Griffiths & Christensen 2007 [48]; MoodGYM + Blue Pages	Systematic review	International	N/A^c,d^	CCBT may be inconsistent with rural residents’ preferred mode of learning—should consider tailoring programs to rural users.
Cheek et al 2014 [46]; SPARX, depression	Qualitative study	Australia	16, 100; male, 75; adolescents, 13-18	New Zealand program acceptable for Australian participants.
Hayward et al 2007 [39]; FearFighter, anxiety & depression	Uncontrolled trial	Scotland	35, 100; female, 66; 16 years and over, 40.2	Participants had significant improvements on measures of depression and anxiety. Patients and GPs^e^were satisfied.
MacGregor et al 2009 [45]; FearFighter, anxiety & depression	Survey & qualitative	Scotland	35, 100; female, 66; 16 years and over, 40.2	Content was generally appropriate for rural dwellers (except for references to city centers, buses, and lifts).
Kay-Lambkin et al 2011; [40] CCBT for comorbid depression & substance use	RCT	Australia	274, 41; male, 57; 16 years and over, 40	Rurality did not affect treatment response (depression). Computerized therapy led to 2.5 times greater reduction in alcohol use than therapist delivered (*P*=.006).
Kay-Lambkin et al 2012 [41]^f^	RCT	Australia	163, 33; N/A; 16 years and over^g^	No significant differences between rural and urban regarding preferred treatment method. No effect of rurality on retention or treatment response.
Mewton et al 2012 [42]; CRUfAD clinic, anxiety	Quasi-experimental	Australia	588, 43; female, 71; 16 years and over, 39.5	Those in a nonrural location were 1.8 times more likely to complete the six course components. Need to tailor courses for rural users.
Sunderland et al 2012 [44]; CRUfAD clinic, depression & anxiety	Quasi-experimental	Australia	663, ~45; female, 66; N/A, 43	Rurality did not influence effectiveness of CCBT for anxiety and depression.

^a^Randomized controlled trial (RCT).

^b^Computerized cognitive behavior therapy (CCBT).

^c^Not applicable (N/A).

^d^The review included 12 papers regarding nine studies. Of these nine studies, five were regarding adults, one regarding tertiary students, and three regarding children/secondary school students. Gender breakdown varied across studies.

^e^General practioners (GPs).

^f^Participants from this study were a subset of Kay-Lambkin et al 2011 [40].

^g^Mean age for this subsample was unavailable.

### Efficacy

Three papers, regarding two trials, reported clinical efficacy data disaggregated by location [40,41,44]. All found no difference in the treatment response to CCBT for depression and anxiety between rural and urban participants. Another study, conducted entirely with rural participants, found that CCBT led to significant improvements in anxiety and depression [39].

One school-based study found that rurality predicted high adherence to CCBT among adolescents, and that higher adherence led to greater reductions in depression and anxiety [38]. However, of the four trials with children/young people, none disaggregated efficacy data by rural/urban location. Furthermore, while the review by Griffiths and Christensen included studies reporting the efficacy of CCBT for both children/young people and adults, none of their included studies disaggregated the data by location [48]. It is, therefore, not possible to identify patterns regarding the interaction of rural location, age, and efficacy from the studies included in this review.

### Uptake and Referral

The included studies did not consistently report rates of uptake, in several cases due to retrospective study designs. The study in rural Scotland found that 24% of people referred to CCBT declined to undertake the treatment [39]. In Kay-Lambkin and colleagues’ trial [40], less than 9% (54) of 617 participants assessed for eligibility refused to participate. However, the rate of uptake in this trial was still only 44% of the original participants assessed—40% were excluded as they did not meet inclusion criteria and a further 7% did not attend their first assessment.

The included systematic review [48] found that, of spontaneous users of the CCBT program MoodGYM worldwide, 20.5% were from rural and remote areas. In further support of its acceptability among rural mental health patients, Kay-Lambkin and colleagues reported that almost half of their sample self-referred to CCBT [40].

General practitioners (GPs) have an important role in connecting patients to CCBT in rural communities. Rural participants are more likely to have been referred to CCBT by their GPs than urban participants—23% versus 2% referred by a GP, respectively (*P*<.001) [40]. Hayward and colleagues’ trial in rural Scotland also relied on GPs to connect patients to CCBT [39]. The study reported that CCBT was highly acceptable among GPs in regard to suitability for provision to rural and remote patients.

### Adherence/Attrition

The included studies reported mixed findings in regard to adherence and attrition rates among rural versus urban participants, though they most commonly reported rural participants to be as likely, if not more likely, to adhere to CCBT treatment.

Two studies with adult participants compared rates of adherence by location. Kay-Lambkin and colleagues found that rurality did not affect retention [41]. In contrast, Mewton and colleagues found that rural participants were significantly less likely to complete CCBT, with urban participants almost twice as likely to complete the full course [42]. The two studies with adolescents that compared adherence outcomes by location both found that rural residence predicted significantly greater adherence to the MoodGYM program [38,43]. Lack of availability of alternative services, greater motivation of supervising staff members, or a preference for health self-management in rural participants are potential explanations for this [38,43,48]. Table 2 shows the efficacy and acceptability outcomes of the studies.

**Table 2:** Efficacy and acceptability outcomes.

Study	Uptake	Adherence	Other acceptability	Clinical effect
Calear et al 2013 [38]	N/A^a^(school based)	Rural had greater adherence (*P*=.01).	N/A	Not disaggregated by location.
Cheek et al 2014 [46]	N/A	N/A	New Zealand program acceptable to rural Australian youth; design important.	N/A
Griffiths & Christensen 2007 [48]	20.5% spontaneous users worldwide rural/remote	N/A	Should consider tailoring content. May not be suitable for learning styles of rural participants.	Both programs examined led to improvements in mental health, knowledge, and attitudes to mental health.
Hayward et al 2007 [39]	89 referred; 13 unsuitable; 21 refused; 55 passwords issued (62%)	26 completed (47% of participants who received passwords)	97% satisfied with help received. GPs feel demos of program could increase referrals by GPs.	Significant improvement in depression and anxiety (*P*<.001).
Kay-Lambkin et al 2011 [40]	617 assessed; 244 unsuitable; 54 refused; 274 randomized (44%); 260 began	86 (33% of starters) received all sessions; 163 (63% of starters) completed 3-month follow-up.	N/A	No significant effect of rurality on effectiveness: depression (*P*=.70) or substance use. Therapist and CCBT equally effective for depression (*P*=.02).
Kay-Lambkin et al 2012 [41]	N/A: 3-month follow-up data	Rurality did not affect attendance or therapeutic alliance.	Rurality did not affect preference for therapist/ computerized delivery. Rural less likely to want more therapist contact—18% vs 48% urban.	Rurality did not influence treatment response.
MacGregor et al 2009 [45]	89 referred; 13 unsuitable; 21 refused; 55 passwords issued (62%)	N/A	Content acceptable to rural/remote participants. Minor changes may be beneficial.	N/A
Mewton et al 2012 [42]	N/A	55.1% completion; rural had poorer adherence (*P*<.05). Urban 1.8 times more likely to complete.	N/A	Significant reduction in anxiety and psychological distress; improved quality of life (WHODAS^b^) (all *P*<.001).
Neil et al 2009 [43]	N/A	Rural had greater adherence: whole sample (*P*=.01), school sample (*P*<.001).	N/A	N/A
Sethi 2013 [47]	103 assessed; 89 eligible and randomized (86%)	100% completed (assume none rural as not reported)	Unsuitable for people with low literacy.	N/A, as location of participants not reported.
Sunderland et al 2012 [44]	N/A: data from completers only	N/A	N/A	Rurality did not influence treatment response: depression (*P*=.83), anxiety (*P*=.77).

^a^Not applicable (N/A).

^b^World Health Organization Disability Assessment Schedule (WHODAS).

### Other Measures of Acceptability

There is some evidence to suggest that, on completion, CCBT was considered to be more acceptable to rural than to urban adult participants. In one study, rural participants were more likely to report that CCBT had helped them with their depression and substance use—92% versus 75% of urban participants [41]. Furthermore, fewer rural CCBT participants reported wanting more face-to-face contact as compared with urban participants—18% versus 48%, respectively. Hayward and colleagues found that 97% of their rural (whole) sample was satisfied with the support provided through CCBT [39].

Studies with both young people and adults found that privacy when accessing mental health services was of great importance to rural participants. For example, Cheek et al [46] found that visibility and confidentiality when accessing services, as well as attitudes of health professionals, were barriers to young people accessing mental health services in a small rural town in Australia. They also found that the opportunity to complete CCBT in private was an appealing feature of the treatment. In another study, two-thirds of the rural adult participants missed therapist contact, and yet two-thirds also felt that the benefits of CCBT included increased autonomy and confidentiality [39].

### Risk of Bias

A risk of bias assessment was conducted for all studies, with the exception of the systematic review. Overall, the risk of bias was moderate. This is consistent with a large review of the broader CCBT evidence by Grist and Cavanagh [26], which found an overall moderate risk of bias across 49 studies. It also established that risk of bias was unlikely to influence effect sizes in the included studies.

Across the RCTs included in this review, the risk of bias was rated as low for two studies [38,40], and moderate for two studies [41,47]. Baseline differences across treatment groups, and between completers and noncompleters of outcome measures, were the primary sources of potential bias. Of the quasi-experimental studies, the risk in one study was unclear due to insufficient information on several variables [44] and moderate in another [45], due to low numbers of participants completing the outcome measurements. The three other studies were rated as likely to be at low risk of bias [39,42,43].

The qualitative study by Cheek and colleagues [46] was rated as at moderate risk of bias. Due to its size and scope, replication of the study in varying locations would be valuable to further understand the generalizability of the findings.

Across the included studies, strict participant selection criteria in several may limit the generalizability of their findings. However, a number of the studies included groups of participants who are otherwise often excluded, such as youth and people with severe symptoms or comorbidities. We believe this goes some way toward balancing this limitation.

A number of studies in the broader CCBT literature have found evidence of a publication bias. Studies reporting negative findings are less likely to be published [26,49,50]. In this review, publication bias is also a real possibility, given that we identified and included only published data.

## Discussion

### Overview

Computerized CBT can be clinically effective for the prevention and treatment of anxiety and depression, and offers a valuable alternative to traditional face-to-face delivery. This may be particularly pertinent to the delivery of services in underresourced and otherwise underserved communities.

### Efficacy and Acceptability

We located 11 studies that begin to identify the feasibility of CCBT in rural and remote communities. Notwithstanding diversity in study designs, participants, software packages, styles, and locations of delivery, the studies indicate that CCBT has equal effects for urban and rural participants. Furthermore, they support the effectiveness of CCBT in real-world rural clinical practice and community settings, with all included trials conducted in school, university, community, or clinical (ie, online mental health or GP clinic) sites.

The included studies indicate that rurality is unlikely to have a negative impact on uptake or adherence. Among the wider CCBT evidence base, low uptake has been identified as a key barrier to implementation, with an average of 12% of participants offered CCBT commencing treatment [26,51,52]. The rates of uptake among several studies included in this review were much higher—44% and 56% in studies by Kay-Lambkin et al [40] and Hayward et al [39], respectively. Importantly, these studies included patients with comorbidities and had minimal inclusion criteria, respectively.

Satisfaction and acceptability are generally high among people who undertake CCBT. Acceptability increases significantly once patients have received a demonstration or have undertaken the treatment [19,41,49,52]. Kay-Lambkin et al showed that treatment preference fulfilment—computerized versus therapist delivered—had a greater impact on adherence for rural versus urban participants [41]. Furthermore, changes in depression were significantly associated with treatment preference fulfilment across their whole sample. Fostering understanding and promoting the credibility of CCBT prior to implementation in rural areas may greatly improve its acceptability, uptake, and reach.

This review provides evidence to support a number of the assumed benefits of CCBT for rural populations, including its ability to overcome barriers that have traditionally limited access to mental health services. For example, studies in both Australia and Scotland found that the ability to complete CCBT privately helped minimize confidentiality concerns and stigma regarding accessing mental health services [39,46]. Furthermore, the delivery of CCBT does not rely on a preponderance of trained therapists, and even guided versions require considerably less therapist time than face-to-face CBT [19,53,54]. Staff who are not trained mental health practitioners are able to provide the guidance required by some CCBT programs without significantly reduced clinical effect [32,40,55,56].

### Opportunities and Challenges for Rural Implementation

Computerized CBT has the potential to complement the inadequate numbers of qualified mental health professionals in rural communities. Implementing CCBT within the existing service landscape as a "first response" treatment may be appropriate. Within such a model, all patients would first be offered CCBT, with therapist time reserved for those who do not respond well, or require further or more intensive therapies [18,34]. This could alleviate pressure on trained therapists and ensure their services are available to those most in need. A study in the United Kingdom [57] found that 19% of participants required referral to a therapist on completion of CCBT. These patients then required, on average, only 3.5—compared with the usual 15—sessions of CBT with a therapist. Combining therapist and computer-delivered CBT has also been shown to be a particularly effective method for treating anxiety and depression among adolescents and similarly reduces the therapist time required to treat each patient [47,58].

More research is needed into the feasibility of delivering CCBT across varying geographical and demographic sites and groups. Understanding local barriers to uptake and adherence, and solutions to these, will be crucial, as they are likely to vary between towns, regions, and countries.

The extent to which content needs to be tailored to rural users’ location and age also requires further study. Computerized CBT packages may benefit from being tailored to more accurately reflect the physical nature of the rural context [42], or in line with different learning styles or education levels [47,48]. However, Cheek and colleagues found that a program developed in New Zealand was acceptable to youth in a rural Australian town [46], suggesting the possibility of translation of CCBT programs across locations without significant alterations.

### Limitations

Despite the use of a systematic methodology, it is possible that some studies have been missed. While some hand searching and pearling was conducted, not every reference list of identified reviews and studies was searched. Furthermore, no unpublished findings were included in the review and it is, therefore, at risk of publication bias.

Inclusion criteria were limited to studies that addressed generalized and social anxiety disorders, depression, or several types of anxiety disorders concurrently. CCBT for individual phobias, posttraumatic stress disorder, and postnatal depression were excluded, although such studies could hold valuable insights to inform the wider application of CCBT. Furthermore, CCBT has been used for 25 different clinical disorders [27], not only mental health conditions [59-62]. To ensure that its full potential is realized, a similar review into the efficacy and acceptability of CCBT for other conditions in rural and remote areas would be valuable.

The studies identified were predominantly Australian, with two from Scotland. The conclusions and recommendations drawn are, therefore, particularly relevant to the Australian context. The authors believe, however, that given the similar challenges faced across the world in providing evidence-based mental health to rural communities, these findings can be expected to be relevant to English-speaking countries more broadly.

### Conclusions

There is a strong focus on workforce development in rural health research and provision. Yet rural and remote communities, globally, continue to face significant challenges in attracting specialist health professionals, highlighting the need for alternative models of delivering evidence-based care. The studies that we reviewed provide initial evidence that CCBT could be a valuable tool for increasing the accessibility of psychological therapies in rural and remote communities. It is likely to be effective and acceptable among rural participants and practitioners.

In the future, practitioners need to be supported to understand and refer clients with particular needs to appropriate evidence-based CCBT programs. Workforce development programs at university level and beyond need to prepare the workforce to appreciate the potential of CCBT. Demonstration of CCBT packages aimed at both users and practitioners may be an important action to build acceptability and trust in rural communities and to ensure that the therapy is accessed by those who need it.

Future research is required to clarify the findings of this review, given the relatively small number of studies identified and the small number of countries represented. Models of CCBT delivery that work within existing health systems and fill service gaps need to be developed and tested in varied rural and remote environments and countries.

## Appendix

Multimedia Appendix 1 Search strategy.

## Abbreviations

CBT: cognitive behavior therapy
CCBT: computerized cognitive behavior therapy
GAD: generalized anxiety disorder
GP: general practitioner
GRADE: Grades of Recommendation, Assessment, Development, and Evaluation
IPT: interpersonal therapy
OCD: obsessive compulsive disorder
PRISMA: Preferred Reporting Items for Systematic Review and Meta-Analysis
RCT: randomized controlled trial
WHODAS: World Health Organization Disability Assessment
