# Supplementary material for: Computerized Cognitive Behavior Therapy for Anxiety and Depression in Rural Areas: A Systematic Review
Source: J Med Internet Res. 2015 Jun 5;17(6):e139. doi: 10.2196/jmir.4145 (PMC4526901; doi:10.2196/jmir.4145)
Supplement: Multimedia Appendix 1 [file jmir_v17i6e139_app1.pdf]

Multimedia Appendix 1

Search strategy, Medline, May 22nd, 2014

1. depression/ or anxiety disorders/ or depressive disorder/
2. "major depression".mp.
3. (depression or "depressive disorder?" or "anxiety disorder?" or anxiety).mp. [mp=title, abstract, original title, name of substance word, subject heading word, keyword heading word, protocol supplementary concept word, rare disease supplementary concept word, unique identifier]
4. 1 or 2 or 3
5. ("mobile health" or e?health or m?health).mp. [mp=title, abstract, original title, name of substance word, subject heading word, keyword heading word, protocol supplementary concept word, rare disease supplementary concept word, unique identifier]
6. (electronic or computeri?ed or online or app or application or mobile).mp. [mp=title, abstract, original title, name of substance word, subject heading word, keyword heading word, protocol supplementary concept word, rare disease supplementary concept word, unique identifier]
7. (health or clinic? or clinical).mp. [mp=title, abstract, original title, name of substance word, subject heading word, keyword heading word, protocol supplementary concept word, rare disease supplementary concept word, unique identifier]
8. 6 and 7
9. 5 or 8
10. Cognitive Therapy/
11. ("cognitive therap$" or CBT or "cognitive?behavio?r therap$" or CCBT or "computeri?ed cognitive?behavio?r therap$").mp. [mp=title, abstract, original title, name of substance word, subject heading word, keyword heading word, protocol supplementary concept word, rare disease supplementary concept word, unique identifier]
12. 10 or 11
13. 4 and 9 and 12
